# Preimplantation genetic testing for Neurofibromatosis type 1: more than 20 years of clinical experience

**DOI:** 10.1038/s41431-023-01404-x

**Published:** 2023-06-19

**Authors:** Vivian Vernimmen, Aimée D. C. Paulussen, Jos C. F. M. Dreesen, Ron J. van Golde, Masoud Zamani Esteki, Edith Coonen, Marianne L. van Buul-van Zwet, Irene Homminga, Alwin A. H. A. Derijck, Lloyd Brandts, Constance T. R. M. Stumpel, Christine E. M. de Die-Smulders

**Affiliations:** 1 GROW-School for Oncology and Reproduction, Maastricht University, Maastricht, The Netherlands; 2 Department of Clinical Genetics, Maastricht University Medical Center, Maastricht, The Netherlands; 3 Department of Obstetrics and Gynecology, Maastricht University Medical Center, Maastricht, The Netherlands; 4 Department of Obstetrics and Gynecology, University Medical Center Utrecht, Utrecht, The Netherlands; 5 University of Groningen, University Medical Center Groningen, Department of Obstetrics and Gynecology, Section Reproductive Medicine, Groningen, The Netherlands; 6 Amsterdam UMC location University of Amsterdam, Center for Reproductive Medicine, Amsterdam, The Netherlands; 7 Amsterdam Reproduction and Development, Preconception and Conception, Amsterdam, The Netherlands; 8 Department of Clinical Epidemiology and Medical Technology Assessment, Maastricht University Medical Center, Maastricht, The Netherlands

**Keywords:** Genetic testing, Genetic counselling, Neurological disorders, Genetic techniques

## Abstract

Neurofibromatosis type 1 (NF1) is an autosomal dominant disorder that affects the skin and the nervous system. The condition is completely penetrant with extreme clinical variability, resulting in unpredictable manifestations in affected offspring, complicating reproductive decision-making. One of the reproductive options to prevent the birth of affected offspring is preimplantation genetic testing (PGT). We performed a retrospective review of the medical files of all couples (*n* = 140) referred to the Dutch PGT expert center with the indication NF1 between January 1997 and January 2020. Of the couples considering PGT, 43 opted out and 15 were not eligible because of failure to identify the underlying genetic defect or unmet criteria for in vitro fertilization (IVF) treatment. The remaining 82 couples proceeded with PGT. Fertility assessment prior to IVF treatment showed a higher percentage of male infertility in males affected with NF1 compared to the partners of affected females. Cardiac evaluations in women with NF1 showed no contraindications for IVF treatment or pregnancy. For 67 couples, 143 PGT cycles were performed. Complications of IVF treatment were not more prevalent in affected females compared to partners of affected males. The transfer of 174 (out of 295) unaffected embryos led to 42 ongoing pregnancies with a pregnancy rate of 24.1% per embryo transfer. There are no documented cases of misdiagnosis following PGT in this cohort. With these results, we aim to provide an overview of PGT for NF1 with regard to success rate and safety, to optimize reproductive counseling and PGT treatment for NF1 patients.

## Introduction

Neurofibromatosis type 1 (NF1) is a hereditary disease with a prevalence of approximately 1:2500–3000 [1–3]. It is a neurocutaneous disorder that mainly affects the skin and the nervous system, with characteristics such as café-au-lait macules, freckling, neurofibromas, and brain and peripheral nerve tumors with a risk of malignancy. Cognitive and psychosocial problems, including learning disabilities, attention-deficit/hyperactivity disorder, and autism spectrum disorders, are more prevalent in the NF1 population compared to the general population [1, 4]. Vasculopathy, such as hypertension and vascular abnormalities of the heart, brain, kidneys, or other major arteries, is an important cause of complications or even early death in patients with NF1 [4]. The life expectancy is about 10–15 years shorter than the general population and lifelong medical follow-up is advised, preferably by a specialized NF1 clinic [5, 6].

NF1 is an autosomal dominant condition caused by mutations in Neurofibromin 1 *(NF1)*, a large gene consisting of 57 exons. The mutation rate is high, without a clear hot spot, resulting in a 50% de novo occurrence and many different reported variants scattered across the gene [2, 3]. The risk of transmission to offspring is 50%. NF1 is fully penetrant with extremely variable clinical manifestations, even within families carrying the same variant. A clear genotype-phenotype correlation has only been established for a few *NF1* variants and remains an area of active research. Consequently, the manifestations of NF1 in affected offspring are mostly unpredictable [7, 8].

The unpredictable clinical expression in offspring can complicate reproductive decision-making for NF1 patients and their partners [9–12]. One of the options for preventing the birth of an affected child is invasive prenatal diagnostics (PND) by chorionic villus sampling (CVS) or amniocentesis with the option to terminate the pregnancy if the fetus is affected. Another option is preimplantation genetic testing (PGT). In PGT, embryos generated through in vitro fertilization (IVF) are tested for the genetic condition and unaffected embryos are transferred to the uterus [13]. Invasive PND is offered to all couples with an ongoing pregnancy after PGT as recommended by the current guidelines due to the small risk of misdiagnosis [14–17].

Currently, literature on PGT for NF1 is limited and primarily focused on the technical aspects [18–21] or single case reports [22, 23]. Only one publication describes a larger number of patients including data on PGT cycles [24]. Previously, an increased risk of various pregnancy-related complications was reported for women with NF1 [25–27]. However, literature on possible complications of IVF treatment in women with NF1 is lacking.

Our aim is to give a complete and detailed overview of PGT for NF1 in a large European PGT center. We focused on pregnancy rate and live birth rate, as well as on preconception screening and IVF treatment, which are previously under-reported aspects of PGT treatment for couples with NF1. Our definitive goal is to optimize reproductive counseling and PGT treatment for both women and men affected with NF1.

## Materials and methods

### Population

The study cohort consisted of couples considering PGT because of NF1 who were referred to the only PGT center in the Netherlands (Maastricht University Medical Center + ) between January 1997 and January 2020. All couples (*n* = 140) were scheduled for counseling with a clinical geneticist prior to PGT treatment. Couples opting for PGT were routinely evaluated by one of the four affiliated IVF (transport) centers (Maastricht University Medical Center+, University Medical Center Utrecht, Amsterdam UMC, University Medical Center Groningen). The PGT cycles were performed in one of these IVF centers. In general, three PGT cycles with oocyte retrieval are reimbursed by the Dutch healthcare system.

### Data collection

Data were collected by reviewing the electronic patient files. Additional data on IVF intake and treatment with possible complications were provided by the IVF centers. Information on the pregnancies and children born after PGT was derived from questionnaires which are routinely sent to the parents after birth. These questionnaires are designed for the general PGT population and therefore do not focus on NF1-specific information.

Data on PGT cycle(s) and embryo transfer(s) were included until December 2020. When PGT treatment resulted in pregnancy during this period, information on the outcomes of those pregnancies was included until the due dates.

### PGT work-up and test development

PGT test development and analysis of the embryos during the PGT treatment for all couples was performed at Maastricht University Medical Center + . PCR protocols employed multiple polymorphic markers flanking the NF1 gene. The number of markers by which the risk and non-risk haplotypes were determined ranged from 3 to 8, using DNA samples from the couple and family members as reference. In case of de novo mutations or unavailable (affected) family members, the specific causative variant was included in the test. Phasing of the risk haplotype in male de novo cases was performed prior to the PGT cycle on the basis of single sperm cell analysis. In the case of female de novo mutations, the phase was determined in the first PGT cycle. For one couple, a next-generation sequencing (NGS) based genome-wide haplotyping (OnePGT) was applied [28–30]. All protocols were designed and validated according to the European Society of Human Reproduction and Embryology guidelines [31]. Specific protocol data are available upon request.

### In vitro fertilization procedures

All couples requesting PGT underwent clinical evaluation in one of the affiliated IVF centers. Work-up consisted of a fertility assessment including vaginal ultrasound of the uterus and ovaries, hormonal testing for women, and sperm analysis for men. Currently, cardiovascular evaluation prior to IVF treatment for women with NF1 is advised.

Following controlled ovarian hyperstimulation combined with a long agonist protocol, oocyte retrieval was performed with subsequent fertilization of oocytes using intracytoplasmic sperm injection. On day 3 or day 5 post-fertilization, eligible embryos were biopsied. After genetic diagnosis, one unaffected embryo was transferred. In women over 37 years, the transfer of two unaffected embryos (if available) was considered. Any remaining unaffected embryos of good quality were cryopreserved for (possible) future transfers.

### Statistical analysis

All statistical analyses were performed using IBM SPSS Statistics 25. The chi-squared test or Fisher’s Exact Test was applied for the comparison of categorical variables between two groups where appropriate. Binary logistic regression analyses were applied to assess the relationship of different variables with the pregnancy rate (defined as an ongoing pregnancy beyond 12 weeks of gestational age). Results were presented by Odds Ratios (OR) and corresponding 95% confidence interval (95% CI). The independent variables included the gender of the affected person (male/female), number of cycles with oocyte retrieval (continuous), number of unaffected embryos available (continuous), and age of the woman at the first cycle with oocyte retrieval (in years, continuous).

## Results

### Participants

**Fig. 1:**
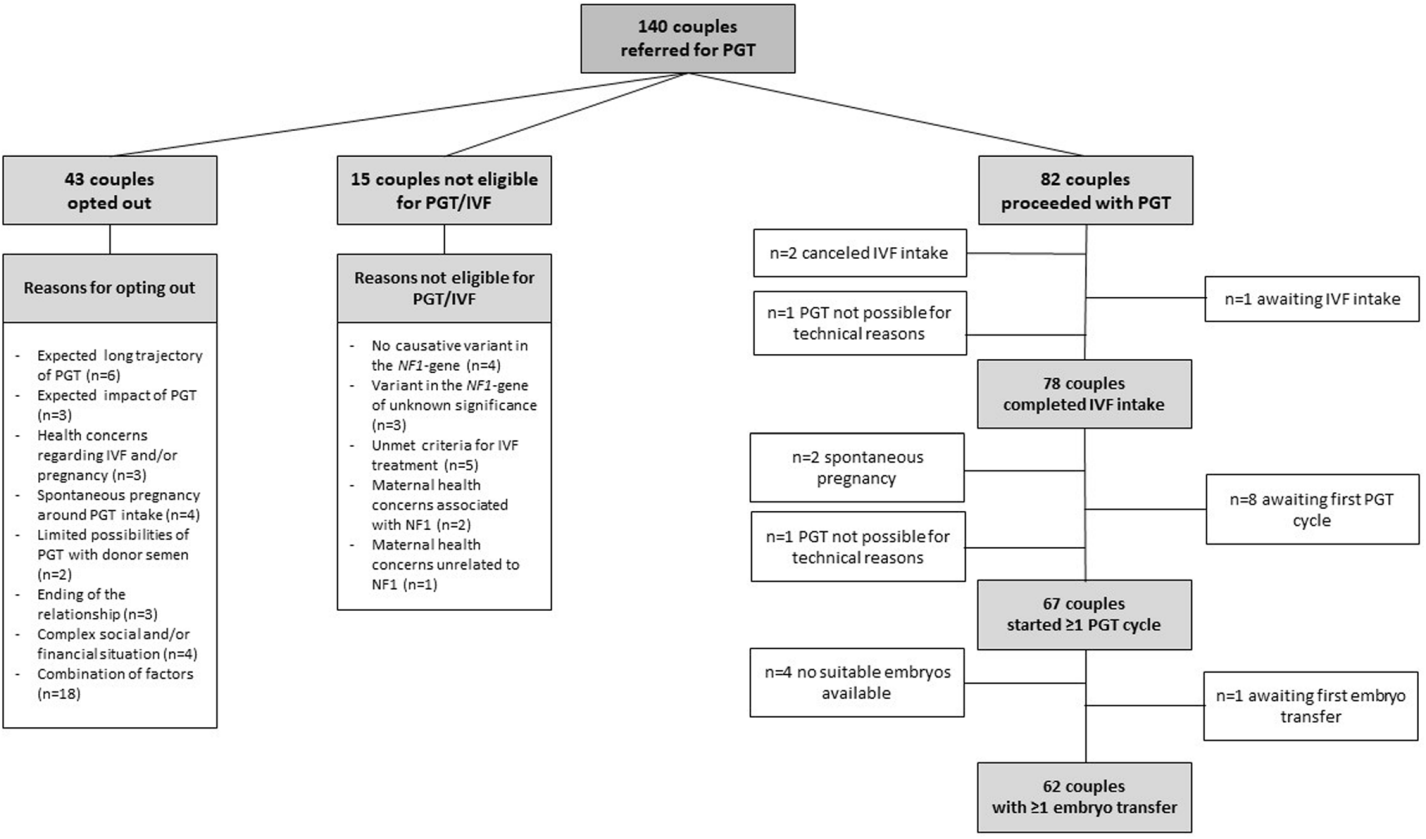
Flowchart showing the referred couples with the indication NF1 in the preimplantation genetic testing process.

Of the total number of couples (*n* = 140), 43 couples (30.7%) opted out after referral for a variety of reasons, as shown in Fig. 1. For 12 couples, the reason involved the PGT procedure, such as the expected long trajectory, emotional impact or maternal health concerns related to the IVF treatment and/or pregnancy. The female was affected in all the couples who refrained because of the expected long trajectory (mean maternal age 32 years, range 27–37 years) or maternal health concerns. Other reasons to refrain were spontaneous pregnancy around the time of PGT intake, limited possibilities of PGT with donor semen, relationship termination, and complex social and/or financial situations. For 18 couples, a combination of factors was presumed to influence the decision to refrain.

Fifteen couples (out of 140, 10.7%) were denied PGT treatment. For 12 couples, the reasons included: the molecular cause of NF1 could not be identified, the variant was classified as of unknown significance, or they did not meet the criteria for IVF treatment (maternal age >40 years at the (expected) start of PGT treatment and/or diminished ovarian reserve). In three cases, PGT treatment was denied by the clinician because of maternal health concerns: one woman affected with NF1 had recently been diagnosed with breast cancer and a second woman suffered from severe NF1-associated vascular problems (stenosis of the aorta and the renal arteries). The third case involved a woman unaffected by NF1 that had an ovarian malignancy. PGT test development was initiated for the remaining 82 couples.

### Baseline characteristics and previous reproductive history

Baseline characteristics and previous reproductive history for these 82 couples are shown in Table 1. In the majority of cases (58.5%), the female was the affected partner. More than half of the cases were (presumed) sporadic (63.4%). 18 couples had a spontaneous pregnancy prior to PGT intake (22.0%) with a total of 30 pregnancies. In 11 of these, no invasive PND was performed, resulting in the birth of eight affected children. Invasive PND was performed in 14 spontaneous pregnancies, showing an affected fetus in 12 cases. Couples opted for termination in all affected pregnancies. The remaining five pregnancies resulted in a miscarriage. One couple underwent previous IVF treatment without PGT in a non-affiliated IVF center. They became aware of the PGT option only after an affected child was born.

**Table 1:** Baseline characteristics and previous reproductive history of the couples proceeding with PGT (*n* = 82).

**Gender affected partner**	***n*** **(%)**	
Male	34 (41.5)	
Female	48 (58.5)	
**Age at PGT intake**	**Mean in years (*n*)**	**Range in years**
Age male	33 (81)	24–52
Age female	29 (82)	22–38
**Inheritance mutation**	***n*** **(%)**	
Sporadic	46 (56.1)	
Presumed sporadic	6 (7.3)	
Familial	30 (36.6)	
**Previous reproductive history**	***n*** **(%)**	**Range per couple**
Couples with spontaneous pregnancy	18 (22.0)	
Number of pregnancies	30	1–5
Miscarriage	5 (16.7)	
Pregnancies without invasive PND	11 (36.7)	
Pregnancies with invasive PND	14 (46.7)	

*PGT* preimplantation genetic testing, *PND* prenatal diagnosis.

### Fertility and preconception screening

Results of fertility and preconception screening are shown in Table 2. Fertility screening showed no signs of sub- or infertility in the majority of the couples (70.6% and 77.3%, male- and female-affected, respectively). The rate of abnormal semen analysis seemed higher in affected males (20.6% versus 6.8%, *p* = 0.09). About two-thirds of the women were referred for cardiac evaluation prior to the start of IVF treatment (65.9%). Evaluation by a cardiologist revealed abnormalities in five cases (17.2%), including hypertension and mitral valve prolapse and/or insufficiency. None of these findings were considered a contraindication for IVF treatment or pregnancy. The comprehensive vascular screening was performed in five women with NF1 (11.4%) because of additional risk factors for vascular complications, such as obesity or a family history of cardiovascular complications (data not shown). A risk profile for vascular complications, including placental insufficiency and preeclampsia, was identified in three of the five women (60.0%) (data not shown).

**Table 2:** Fertility and preconception screening for couples completing IVF assessment.

	Male affected	Female affected	*p*-value
	*n* (%)	*n* (%)	
**Couples completing IVF assessment (*n*** = **78)**	34	44	
**Fertility assessment**			
No subfertility or infertility	24 (70.6)	34 (77.3)	
Subfertility or infertility	10 (29.4)	10 (22.7)	
Female factor sub- or infertility (yes, %)	3 (8.8)	6 (13.6)	0.72
Male factor sub- or infertility* (yes, %)	7 (20.6)	3 (6.8)	0.09
Primary sub- or infertility (yes,%)	0 (0.0)	1 (2.3)	
**Cardiovascular evaluation in women with NF1 (*n*** = **44)**			
Cardiac evaluation performed	n.a.	29 (65.9)	
No abnormalities documented		19 (65.5)	
Abnormalities documented		5 (17.2)	
Result missing		5 (17.2)	
No cardiac evaluation performed	n.a.	15 (34.1)	

*IVF* in vitro fertilization, *NF1* Neurofibromatosis type 1, *n.a.* not applicable.

*Oligo- and/or asthenozoo- and/or teratozoospermia necessitating intrauterine insemination (IUI) or intracytoplasmic sperm injection (ICSI).

### Clinical data on performed PGT cycles and pregnancy outcome

Clinical data on performed PGT cycles and pregnancy outcomes are shown in Table 3. In total, 67 couples initiated 143 cycles, 12 of which were canceled because of hypo- or hyperstimulation. Single blastomere biopsy was performed in 106 cycles and trophectoderm biopsy (5–8 cells) in 25 cycles. Of the 131 cycles with oocyte retrieval, 111 resulted in an embryo transfer (ET). Nineteen cycles did not result in an ET because no unaffected embryos of sufficient quality were available. One couple was awaiting their first frozen ET at the time data collection for the study was finalized. A genetic diagnosis (affected or unaffected) could be obtained in 595 of the 746 biopsied embryos (79.8%). The result was inconclusive (because of recombination or apparent contamination) or aberrant (monosomy or trisomy) in 151 (out of 746) embryos (20.2%). The transfer of 174 unaffected embryos resulted in 61 positive HCGs (human chorionic gonadotropin) and 42 ongoing pregnancies (35.1% and 24.1%, respectively) for 37 couples. Of the 37 couples with an ongoing pregnancy, 32 achieved one pregnancy and for five couples PGT treatment(s) resulted in two pregnancies. The overall pregnancy rate was 30.8% after a fresh transfer and 16.9% after transfer of a frozen and thawed embryo. The cumulative pregnancy rate per couple with one or more initiated PGT cycles (*n* = 67) was 62.7% and 67.7% per couple with one or more ET (*n* = 62). There were 42 live births after PGT, including one dichorionic diamniotic twin following a single embryo transfer. The number of unaffected embryos available for ET was positively correlated with the pregnancy rate (*p* = 0.03, OR 1.345, 95% CI 1.04–1.75). Other analyzed variables possibly influencing pregnancy rate (gender of the affected partner, number of cycles with oocyte retrieval, and age of the woman at first ovum pick-up) showed no significant impact on pregnancy rate (data shown in Supplemental Table 1).

**Table 3:** Clinical data on performed PGT cycles and pregnancy outcome (*n* = 67).

Cycles	*n*
Cycles started	143
Cycles to oocyte retrieval	131
Cycles to embryo biopsy	131
Cycles with blastomere biopsy	106
Cycles with trophectoderm biopsy	25
Cycles to embryo transfer	111

Embryo biopsies and analysis	*n* (%)
Embryos biopsied	746
Blastomere biopsy	698
Trophectoderm biopsy	48
Embryos with diagnosis	595 (79.8)
Number of embryos unaffected	295 (49.6)
Number of embryos affected	300 (50.4)
Embryos inconclusive or aberrant	151 (20.2)

Embryo transfers	*n*
Embryos transferred	174
Fresh embryo transfers	91
Frozen embryo transfers	83

Pregnancies and births	*n*
hCG positive	61
Ongoing pregnancies	42
Live birth deliveries	41
Babies	42 (40 singletons, 1 twins)
No congenital anomalies	38
Congenital anomalies	2
Missing	2
Outcome missing	1

Outcome	%
Pregnancy rate	
Per embryo transfer	24.1
Per fresh embryo transfer	30.8
Per frozen embryo transfer	16.9
Live birth rate	
Per embryo transfer	23.6

*PGT* preimplantation genetic testing, *hCG* human chorionic gonadotropin.

Invasive prenatal diagnostics by chorionic villus sampling or amniocentesis were performed in four pregnancies. The familial mutation in the *NF1* gene was not detected in these cases, confirming the PGT analysis result. An amniocentesis was performed in one additional pregnancy because of fetal anomalies observed by ultrasound. The familial mutation in the *NF1* gene was not detected and additional genetic testing applying whole exome analysis revealed no other explanation for the ultrasound abnormalities. The pregnancy was continued. After birth, the diagnosis VACTERL association was made (vertebral defects, anal atresia, cardiac defects, tracheo-esophageal fistula, renal anomalies, and limb abnormalities). The female was the affected partner in all pregnancies in which prenatal diagnostics were performed. Hypospadias was documented in one other live birth and considered unrelated to NF1.

### Clinical data on complications related to IVF treatment and pregnancy

Clinical data on complications related to IVF treatment and pregnancy are shown in Table 4. Complications of IVF treatment (ovarian hyperstimulation syndrome and/or ovarian bleeding) were reported in two (out of 40) women with NF1 (5.0%) and in four (out of 27) women (14.8%) where the male was affected with NF1 (*p* = 0.21).

**Table 4:** Clinical data on complications related to IVF treatment and pregnancy.

	Male affected	Female affected	*p*-value
	*n* (%)	*n* (%)	
**Couples undergoing** ≥ **1 IVF treatment (*n*** = **67)**	27	40	
No complications documented	23 (85.2)	38 (95.0)	
Complications documented	4 (14.8)	2 (5.0)	0.21

	Male affected	Female affected	
	*n* (%)	*n* (%)	
**Number of ongoing pregnancies (*n*** = **42)**	16	26	
No complications documented	15 (93.7)	18 (69.2)	
Complications documented	1 (6.3)	4 (15.4)	
Missing	0 (0.0)	4 (15.4)	
Increase in neurofibromas documented	n.a.	3 (11.5)	

*IVF* in vitro fertilization, *n.a.* not applicable.

Pregnancy complications (hypertension, preeclampsia, and gestational diabetes) were reported in four (out of 26) ongoing pregnancies (15.4%) in females with NF1. An increase in neurofibromas was reported in three pregnancies (11.5%). Hypertension was documented in one (out of 16) pregnancies in a female unaffected with NF1 (6.3%).

### Clinical follow-up after PGT

PGT treatment did not result in an ongoing pregnancy for 18 (out of 67) couples (26.9%). Eight couples ended their PGT treatment prematurely (before three cycles were completed) on their own initiative for a combination of reasons: maternal health (*n* = 1, unrelated to NF1 as the male was affected), death of partner (*n* = 1, related to NF1 in the affected male), the emotional impact of PGT treatment (*n* = 2), the low perceived chance of success as there were no unaffected embryos available for transfer in the previous cycle (*n* = 1, this couple chose IVF without PGT) and a spontaneous pregnancy between cycles (*n* = 1). For two couples, the reason for discontinuing the PGT treatment was unknown. Ten couples completed at least three PGT cycles without achieving an ongoing pregnancy.

## Discussion

We described a cohort of couples requesting PGT for NF1 in a large European center over a period of more than 20 years. We found an ongoing pregnancy rate of 24% (42 out of 174) per ET, which is similar to the reported clinical pregnancy rate per ET of 29% in PGT for autosomal dominant monogenic disorders in the European Society of Human Reproduction and Embryology data registry [32] and the general reported clinical pregnancy rate after PGT of 24% in the Netherlands (PGT annual report 2020, www.pgtnederland.nl). Our live birth rate per cycle is higher than reported for another large cohort (*n* = 77 couples) describing PGT for NF1 in the United States: 31% (41 out of 131) (confirmed) live birth rate per cycle with oocyte retrieval versus 14%, respectively [24]. In the US cohort, experience with PGT for NF1 in the referring IVF center was considered as one of the possible factors that influenced success rates, although no statistically significant differences were identified. This could (partially) explain the difference, since in the US cohort 62 IVF centers were involved compared to 4 in our cohort. Not unexpectedly, the chance of pregnancy in our cohort increased with the number of unaffected embryos available for transfer.

Invasive PND was performed in only five pregnancies (12%, 5 out of 42). All confirmed the PGT results in our cohort. Although the percentage of PND is higher in our cohort than in the cohort receiving PGT treatment for polycystic kidney disease described by Berckmoes et al. (4.3%) [33], 12% still seems low since all couples with an ongoing pregnancy were offered confirmation with invasive PND. However, the fact that most couples did not choose confirmatory PND after PGT has been previously described in the literature [14]. Reasons to refrain from confirmatory (invasive) PND included concerns about the risks of miscarriage and confidence in the accuracy of PGT, especially in patients with less severe or late-onset conditions [14]. Information on postnatal molecular diagnosis is largely lacking in our cohort, but no reports of clinical misdiagnosis have been communicated to the PGT center and/or the affiliated IVF centers.

We found that 31% (43 out of 140) of the referred couples considering PGT for NF1 opted out. PGT was not possible for 11% (15 out of 140) of the couples because of failure to identify the underlying genetic cause, unmet criteria for IVF treatment, or maternal health concerns regarding the IVF treatment. This highlights the importance of timely reproductive counseling and genetic confirmation of the clinical diagnosis of NF1 to make PGT available for also these couples.

In our cohort, the female was affected in the majority of the cases. A similar distribution was described in another cohort reporting on PGT for NF1 [24], but also in cohorts describing PGT for Huntington’s disease [34, 35] and monogenic kidney disease [36]. With genders being equally affected, a possible explanation could be that reproductive counseling gets more attention from women with a hereditary condition compared to men, making women more aware of their reproductive options. Another factor could be the influence of the gender of the affected partner in the reproductive decision-making itself. A qualitative interview study showed that in reproductive decision-making between partners, the woman’s influence is considered greater since she is the individual undergoing the treatment and/or carrying the pregnancy [37]. Furthermore, feelings of guilt towards the (unaffected) partner seem to play a role, especially when the male is the affected partner, since this requires the woman to undergo ovarian hyperstimulation and transvaginal ovum retrieval [37]. This could result in refraining from PGT when the male is affected.

Previous studies have shown reduced reproductive fitness in women and men with NF1, but this has been attributed to social rather than biological factors [38]. Males with NF1 showed a higher percentage of sperm abnormalities compared to the group in which the female was the affected partner. Although the statistical power of this study was insufficient to detect a significant difference, this finding could be clinically relevant and may suggest reduced fertility in men with NF1. Moreover, a study has shown altered spermatogenesis and reduced fertility in a NF1 mouse model due to haploinsufficiency of neurofibromin [39]. As infertility due to insufficient sperm motility and/or concentration may already necessitate IVF treatment, the higher percentage of sperm abnormalities found in the current PGT cohort of NF1-affected men may reflect a reduced threshold to add PGT to an already existing need for IVF. However, not only known, but also previously unknown male infertility was more prevalent in the NF1 couples where the male was affected. This supports that not only bias but also a biological factor related to NF1 may play a role.

One patient could not undergo IVF treatment due to previously known major NF1-related cardiovascular problems. Cardiac evaluation in the other women with NF1 showed some cardiovascular problems, but those were not considered a contraindication for IVF treatment or pregnancy. These observations raise the question of whether cardiac evaluation is warranted in all women with NF1 prior to IVF treatment, or only in those cases with clinical signs indicating or previously known cardiovascular problems.

Complications of IVF treatment were reported in some women with NF1, but not more frequently than in the couples where the male was affected with NF1. These results suggest that IVF treatment in women with NF1 does not translate into a higher rate of complications. The rate of ovarian hyperstimulation syndrome observed in our cohort was higher than reported in the literature (7.5%, 5 out of 67, versus 0.5–5% respectively). This may be due to the small number of our cohort and the fact that the reported prevalence in literature mainly concerns the severe form of ovarian hyperstimulation syndrome [40]. Furthermore, as the use of short antagonist protocols during hyperstimulation increases, the risk of ovarian hyperstimulation syndrome will decrease.

Previous studies have shown that vascular pregnancy-associated complications such as gestational hypertension and preeclampsia are more prevalent in women with NF1. The percentage of (gestational) hypertension and preeclampsia in our cohort (11.5%, 3 out of 26) is similar to a large cohort of NF1 cases from the United States (11.3%) [26] which is higher than the average global incidence (4.6%) [41]. There were no reported cases of acute cardiovascular events, stillbirth, or maternal deaths in our cohort. Although the numbers are small, we found no major adverse events during pregnancy, which corresponds to the finding of Terry et al. in a cohort of pregnant women with NF1 [26]. There have been some case reports on malignant peripheral sheath tumors or large plexiform neurofibromas in women with NF1 during pregnancy [42–45]. We found no such events in our cohort.

Our study has limitations due to its retrospective design and population size. However, the population size is similar to other studies reporting on PGT for a specific indication [24, 33, 36] and given the two decades time period this study has covered, the feasibility of a larger prospective study seems very limited. There are also several strengths to this study. We were able to include all referred couples with the indication NF1 to our PGT expert center and performed an in-depth assessment of the patient files, resulting in a complete and detailed overview. Thereby, we provide valuable, disease-specific information which could aid future reproductive counseling of NF1 patients worldwide.

PGT is a successful reproductive option for preventing the birth of NF1-affected offspring. Although acknowledging the limitations of our study, we did not find a clinically or statistically significant difference in IVF complications in our cohort when comparing NF1-affected females to partners of affected males. Cardiac screening in women with NF1 prior to IVF treatment showed no (previously unknown) contraindications, raising the question of whether this diagnostic step is necessary. Timely reproductive counseling is important. For several couples, PGT was not possible due to factors such as maternal age and/or diminished ovarian reserve or the inability to identify an underlying genetic defect. Also, we observed a non-significant increased rate of male infertility in men affected with NF1, knowledge which could aid couples in opting for adding PGT to an already indicated IVF treatment.

In particular for NF1 patients, reproductive choices are challenging, mostly due to the extreme variability of the condition. PND with the possible termination of a pregnancy is often a difficult choice due to the potential of mild features in offspring. However, PGT is often motivated by a fear of a severe NF1 phenotype in offspring. We will further study these psychosocial aspects in future research projects. The challenges related to NF1 encountered during PGT test development, such as the high rate of sporadic mutations and the occurrence of mosaicism in the affected person, will also be addressed in a future research project.

## Supplementary information

Supplemental material 1

## Data Availability

The data underlying this article cannot be shared publicly due to the privacy of the individuals that participated in the study. The data will be shared on reasonable request to the corresponding author.
