# Supplementary material for: Preimplantation genetic testing for Neurofibromatosis type 1: more than 20 years of clinical experience
Source: Eur J Hum Genet. 2023 Jun 19;31(8):918–24. doi: 10.1038/s41431-023-01404-x (PMC10400537; doi:10.1038/s41431-023-01404-x)
Supplement: Supplementary file 1 — Supplemental material 1 [file 41431_2023_1404_MOESM1_ESM.docx]

| **Supplemental material - Table I. Variables possibly influencing pregnancy rate**  **(couples n=66, events defined as first pregnancy n=37)** | | | |
| --- | --- | --- | --- |
|  | **OR** | **95% CI** | **p-value** |
| **Male affected** | Reference |  |  |
| **Female affected** | 1.00 | 0.34-2.94 | 1.00 |
| **Number of cycles with oocyte retrieval (per 1 increase)** | 0.88 | 0.48-1.60 | 0.67 |
| **Number of unaffected embryos available for transfer (per 1 increase)** | 1.35 | 1.04-1.75 | 0.03 |
| **Age woman at first cycle with oocyte retrieval (per 1 year increase)** | 0.95 | 0.84-1.08 | 0.45 |
